# Vaginal sparing in laparoscopic radical cystectomy for females: feasibility and technical notes

**DOI:** 10.1590/S1677-5538.IBJU.2019.0021

**Published:** 2020-09-02

**Authors:** Cinthia Alcántara-Quispe, Eliney Ferreira Faria, Alexandre Cesar Santos, Wesley Justino Magnabosco, João Fantin, Marcos Tobias-Machado, Roberto Dias Machado

**Affiliations:** 1 Hospital de Cancer de Barretos BarretosSP Brasil Hospital de Cancer de Barretos - Urologia Barretos, Barretos, SP, Brasil

## INTRODUCTION

Anterior pelvic exenteration with urinary diversion (UD) and extended lymphadenectomy is the standard procedure for invasive tumors of the bladder in women ( 1 ), resulting in 52% of female sexual dysfunction after the procedure ( 2 ). Less radical techniques with pelvic organ preservation are viable alternatives in selected cases such as sexually active women with early-stage neoplasms ( 3 ). Although still with preliminary data, these techniques seem to respect the foundations of the TRIFECTA principle ( 2 ). The objective of this video is demonstrate a laparoscopic surgical technique used by our team for the vaginal and sphincter complex preservation during radical cystectomy (RC) with UD.

## CASE REPORT

A 50-year-old female patient who was sexually active, and diagnosed with pT2 high-grade urothelial carcinoma (UC) associated with carcinoma in situ, located in the anterior wall bladder. She underwent laparoscopic RC and orthotopic UD with preservation of the lower three-fourths of the vaginal duct and urethral sphincter complex. The surgical extirpating time was 180min, with and estimated 400mL of bleeding. The pathological finding revealed high-grade UC with free margins. She evolved without serious complications, early diurnal urinary continence, and vaginal intercourse at 5 months after the procedure.

## CONCLUSIONS

Vaginal preservation by minimally invasive techniques is a real option with positive impact on the quality of life with better urinary and sexual functional outcome when compared with other radical techniques. Although robotic surgery is currently gaining ground in this scenario, laparoscopic surgery is still a viable option in the Robotic era, in places of low income.
